# The Tumor Suppressor TFF1 Occurs in Different Forms and Interacts with Multiple Partners in the Human Gastric Mucus Barrier: Indications for Diverse Protective Functions

**DOI:** 10.3390/ijms21072508

**Published:** 2020-04-04

**Authors:** Jörn Heuer, Franziska Heuer, René Stürmer, Sönke Harder, Hartmut Schlüter, Nayara Braga Emidio, Markus Muttenthaler, Dörthe Jechorek, Frank Meyer, Werner Hoffmann

**Affiliations:** 1 Institute of Molecular Biology and Medicinal Chemistry, Otto-von-Guericke University Magdeburg, Leipziger Str. 44, 39120 Magdeburg, Germany; 2 Institute of Clinical Chemistry and Laboratory Medicine, University Medical Center Hamburg-Eppendorf, Martinistr. 52, 20246 Hamburg, Germany; 3 Institute for Molecular Biosciences, The University of Queensland, Brisbane, Queensland 4072, Australia; 4 Institute of Biological Chemistry, Faculty of Chemistry, University of Vienna, 1090 Vienna, Austria; 5 Institute of Pathology, Otto-von-Guericke University Magdeburg, 39120 Magdeburg, Germany; 6 Department of Surgery, Otto-von-Guericke University Magdeburg, 39120 Magdeburg, Germany

**Keywords:** gastric cancer, reactive oxygen species, inflammation, trefoil factor, TFF1, FCGBP, gastrokine, lectin, mucin, innate immunity, gastric protection

## Abstract

TFF1 is a protective peptide of the Trefoil Factor Family (TFF), which is co-secreted with the mucin MUC5AC, gastrokine 2 (GKN2), and IgG Fc binding protein (FCGBP) from gastric surface mucous cells. *Tff1*-deficient mice obligatorily develop antropyloric adenoma and about 30% progress to carcinomas, indicating that Tff1 is a tumor suppressor. As a hallmark, TFF1 contains seven cysteine residues with three disulfide bonds stabilizing the conserved TFF domain. Here, we systematically investigated the molecular forms of TFF1 in the human gastric mucosa. TFF1 mainly occurs in an unusual monomeric form, but also as a homodimer. Furthermore, minor amounts of TFF1 form heterodimers with GKN2, FCGBP, and an unknown partner protein, respectively. TFF1 also binds to the mucin MUC6 in vitro, as shown by overlay assays with synthetic ^125^I-labeled TFF1 homodimer. The dominant presence of a monomeric form with a free thiol group at Cys-58 is in agreement with previous studies in *Xenopus laevis* and mouse. Cys-58 is likely highly reactive due to flanking acid residues (PPEEEC^58^EF) and might act as a scavenger for extracellular reactive oxygen/nitrogen species protecting the gastric mucosa from damage by oxidative stress, e.g., H_2_O_2_ generated by dual oxidase (DUOX).

## 1. Introduction

The secretory peptide TFF1 (formerly: pS2) is a member of the Trefoil Factor Family (TFF) [1,2,3]. The major expression site is gastric surface mucous cells [4,5]. This explains why TFF1 is a typical constituent of the gastric juice (about 70 ng/mL) [4], where it is co-secreted together with the mucin MUC5AC, gastrokine 2 (GKN2), and IgG Fc binding protein (FCGBP) [6]. Minor amounts of TFF1 are also secreted by the major and minor salivary glands [7].

Pathologically, TFF1 is ectopically expressed during inflammatory conditions, such as duodenitis, Crohn’s disease, ulcerative colitis, diverticulitis, pancreatitis, and cholecystitis [2,8,9]. Furthermore, TFF1 expression occurs in certain premalignant conditions as well as in various tumors, and TFF1 is used as a prognostic marker, particularly for breast cancer [1,10]. Of note, in approximately 50% of human gastric carcinomas, TFF1 expression is lost [11,12]. Numerous in vivo and in vitro studies point to TFF1 as a key player in mucosal protection, even with clinical perspectives [13].

Inactivation of *Tff1* in mice (*Tff1^KO^*) predominantly results in a gastric phenotype, where all *Tff1^KO^* animals develop antropyloric adenoma with ~30% progressing to carcinoma [11]. Carcinogenesis is age-dependent and follows a sequence of morphological changes that include hyperplasia, low-grade dysplasia, high-grade dysplasia, and invasive adenocarcinoma [14]. This multi-step carcinogenesis is increasingly associated with NF-κB-mediated chronic inflammation [14] and tumor growth can be suppressed with the selective Cox-2 inhibitor celecoxib [12,14,15].

In line with these observations, the continuous self-renewal of the gastric mucosa from stem and precursor cells is dysregulated in *Tff1^KO^* mice [12]. In fundic units, there is an amplification of surface mucous cells at the expense of parietal cells [16]. This situation was even more pronounced in antral units, where the mucosa was thickened and non-functional, producing very little mucus as the amplified cells were progenitor cells [11,17]. Collectively, all these date indicate that *TFF1* is a gastric tumor suppressor gene [12]. However, the question arises as to the molecular function of TFF1.

In the past, the biological function of TFF1 was mainly attributed to its rather weak motogenic effect in vitro (chemotactic factor) [1,2,3,10,18,19,20,21,22,23]. This activity together with an anti-apoptotic effect [24] would be capable of enhancing the rapid repair of the gastric mucosa after damages by cell migration, a process called “restitution” [25,26]. Notably, the weak motogenic effect of TFF1 appears to be in a concentration range of about 10^−6^ to 10^−7^ M [19,21]. This relatively high concentration is atypical of classical peptide ligands (such as epidermal growth factor), which activate their corresponding receptors even at concentrations of 10^−10^ M [27]. It is thus not surprising that the many attempts to characterize a specific receptor for TFF1 or other TFF peptides have failed [28].

Furthermore, homodimeric TFF1 has lectin activities enabling pH-dependent binding to a lipopolysaccharide of *Helicobacter pylori* [29]. This led to the hypothesis that TFF1 plays a role in mediating the tropism of *H. pylori* within the gastric mucus [30].

However, TFF1 has clear protective effects in different animal models of intestinal damage in vivo when overexpressed in transgenic mice or delivered by genetically modified *Lactococcus lactis* [31,32]. Furthermore, a formulation of *L. lactis* secreting TFF1 (AG013) has successfully been tested in a hamster model as well as a clinical phase 1b study to reduce oral mucositis after radiation or chemotherapy [33,34].

To further clarify the molecular function of TFF1, different forms of TFF1 were characterized in various studies. The 60-residue peptide TFF1 contains seven cysteine residues, with three intramolecular disulfide bridges stabilizing the conserved TFF domain [1]. Recombinant TFF1 produced in *Escherichia coli* forms homodimers via Cys-58 that is situated outside the TFF domain [35]. In gastric mucosa, in addition to faint amounts of TFF1 homodimer and monomer, a predominant TFF1 complex with a relative molecular mass (M_r_) of 25k was described [36], which was later identified as a disulfide-linked heterodimer with GKN2 (formerly: TFIZ1, GDDR) [37]. However, the majority of TFF1 and TFF1-GKN2 is definitely not associated with mucins [38]. Recently, the orthologs of TFF1 (i.e., xP1 and Tff1) were characterized biochemically in the stomach of *Xenopus laevis* and the mouse, respectively [39,40]. Surprisingly, both xP1 and Tff1 occurred to a large extent in their monomeric forms. This is unusual as cysteine residues in secretory proteins are normally oxidized to disulfide bridges [41]. Thus, here, we systematically investigated the different forms of TFF1 in human gastric mucosa using anion-exchange and size exclusion chromatography (SEC), and also performed binding studies with synthetic TFF1. This is a further step towards understanding the molecular function of TFF1, particularly as a gastric tumor suppressor.

## 2. Results

### 2.1. Characterization of Human Gastric Extracts by SEC and Western Blot Analysis

**Figure 1:**
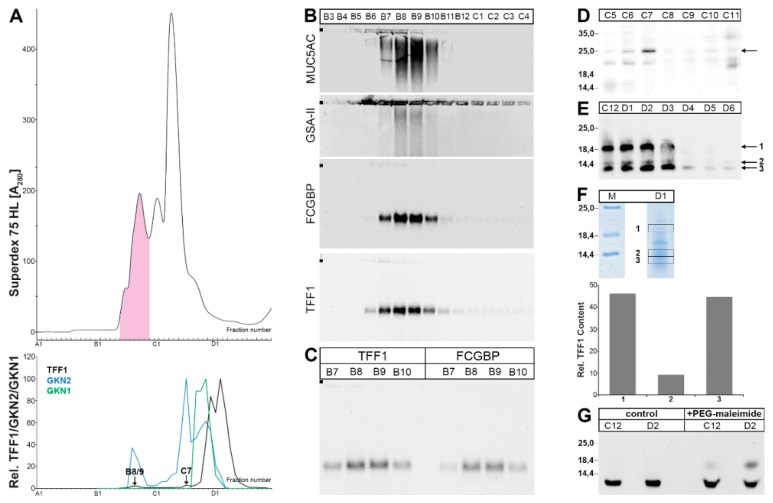
Analysis of human gastric corpus extract (M_C_-383; homogenized in a mortar). (**A**) Elution profile after size exclusion chromatography (SEC) on a Superdex 75 HL column, as determined by absorbance at 280 nm (periodic acid-Schiff (PAS)-positive mucin fractions: pink). Underneath: Distribution of the relative TFF1 (black), GKN2 (blue), and GKN1 (green) content as determined by Western blot analysis under reducing conditions and semi-quantitative analysis of the typical monomeric band intensities. (**B**) 1% agarose gel electrophoresis (AgGE) and subsequent Western blot analysis of the fractions B3-C4 concerning MUC5AC, MUC6 (lectin GSA-II), FCGBP, and TFF1. The start is marked with a dot on the left. (**C**) 1% AgGE and subsequent Western blot analysis of the fractions B7-B10 concerning TFF1 and FCGBP, respectively. (**D**) 15% sodium dodecyl sulfate-polyacrylamide gel electrophoresis (SDS-PAGE) (non-reducing conditions) and subsequent Western blot analysis of fractions C5-C11 concerning TFF1 (post-reduction step on the membrane). The molecular mass standard is indicated on the left. (**E**) 15% SDS-PAGE (non-reducing conditions) and subsequent Western blot analysis of fractions C12-D6 concerning TFF1 (post-in-gel-reduction). (**F**) 15% SDS-PAGE (non-reducing conditions) and Coomassie staining of fraction D1 (M: marker). Then, proteins were eluted from the bands 1, 2, and 3, subjected to reducing 15% SDS-PAGE, and the TFF1 content was measured by semi-quantitative Western blot analysis. (**G**) 15% SDS-PAGE (reducing conditions) and Western blot analysis concerning TFF1 of fractions C12 and D2 with or without polyethylene glycol (PEG)-maleimide treatment.

Human gastric extracts were separated by SEC, and the immunoreactivities for TFF1 and the gastrokines GKN2 and GKN1 (for comparison) were tested (Figure 1), the latter peaking in fractions C10-C12 (Figure 1A, lower panel). TFF1 immunoreactivity appeared in three different regions, to very different extents (Figure 1A). (i) Little TFF1 immunoreactivity was detectable in the periodic acid-Schiff (PAS)-positive high-molecular-mass peak typical of mucins (maximum at fractions B8/B9; Figure 1A). The corresponding band after agarose gel electrophoresis (AgGE; Figure 1B) was congruent with that of FCGBP, indicating the existence of a TFF1-FCGBP heterodimer (Figure 1C). In this region, GKN2 immunoreactivity was also detectable (Figure 1A). (ii) Some TFF1 immunoreactivity was also traceable in fraction C7 (Figure 1A) as a 25k band after non-reducing sodium dodecyl sulfate-polyacrylamide gel electrophoresis (SDS-PAGE; Figure 1D). Here, the major amount of GKN2 immunoreactivity was also present (Figure 1A). Thus, the 25k band is expected to represent the TFF1-GKN2 heterodimer. (iii) The bulk of TFF1 immunoreactivity was present in the low-molecular-mass fractions C11-D3 (Figure 1A) as three bands (18k, 14k, <14k; non-reducing SDS-PAGE; Figure 1E). All three bands contained TFF1, which can be released by reduction (Figure 1F). In this region, little GKN2 immunoreactivity was detected (Figure 1A, lower panel).

To test whether the three low-molecular-mass entities (bands 1–3; Figure 1E) contained a free thiol group, fractions C12 and D2 were treated with polyethylene glycol (PEG)-5000-maleimide and then analyzed under reducing conditions for TFF1 immunoreactivity (Figure 1G). Clearly, part of the TFF1 immunoreactivity was shifted after PEG-maleimide treatment, indicating the presence of free thiols.

### 2.2. Stepwise Extraction of TFF1, GKN2, GKN1, and Mucin from Human Gastric Mucosa

The gastric mucus is composed of two layers, a loosely adherent outer layer contacting the gastric juice and a firmly attached water-insoluble inner layer [42]. As TFF1 is part of both the gastric mucus and the gastric juice, it was tested as to whether TFF1 could be extracted simply by washing the gastric specimens (corpus and antrum, respectively) in a rotating wheel (for details, see Section 4.2.). The supernatant E0 represents an easily diffusible fraction of extracellular proteins. The remaining cell pellet was extracted with buffer yielding supernatant E1, which represents a fraction of soluble proteins. Finally, the remaining cell pellet was extracted twice with 1% SDS (at 50 and 100 °C, respectively) and the combined supernatants E2 were obtained representing a fraction of hardly soluble proteins. In all samples (E0, E1, E2 of the gastric corpus and antrum, respectively), the relative TFF1, GKN2, and GKN1 contents were determined (by semi-quantitative Western blot analysis) as well as the mucin content (by PAS staining).

**Figure 2:**
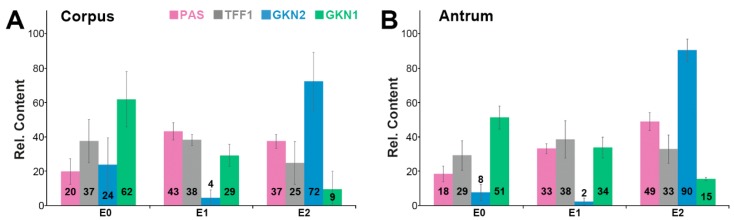
Stepwise extraction of three different human gastric (**A**) corpus and three different (**B**) antrum specimens respectively, and analysis of the extracts E0, E1, and E2. Shown are the relative contents of TFF1 (grey), GKN2 (blue), and GKN1 (green) respectively, as determined by Western blot analysis under reducing conditions and semi-quantitative analysis of the typical monomeric band intensities. For comparison, the mucin content was determined by PAS staining (dot blot; pink).

Generally, the results from the corpus and the antrum were comparable (Figure 2). Clearly, TFF1 could be directly washed out from the specimens (fractions E0) to about 37% (corpus) and 29% (antrum). In the soluble fraction E1, about 38% of TFF1 was present. In the hardly soluble fraction E2, about 25% and 33% of the TFF1 were present.

In contrast, GKN2 was found in the washings E0 to 24% and 8%, and in the soluble fractions, E1 to only 4% and 2%, whereas 72% and 90% remained in the hardly soluble fraction E2.

GKN1 was easily washed out (62% and 51% in E0) or was soluble (29% and 34% in E1). Only a minority was detected in the hardly soluble fraction E2 (9% and 15%).

For mucin, about 20% and 18% could be directly washed out (E0) and 43% and 33% were soluble (E1), whereas 37% and 49% of the mucin were hardly soluble.

### 2.3. Characterization of Human Gastric Extracts after TRIzol^®^ Extraction and SEC

As relatively little TFF1-GKN2 was detectable after classical extraction with aqueous buffer (Figure 1D) and a considerable part of TFF1 and the major amount of GKN2 were found to be hardly soluble (fraction E2, Figure 2), we extracted gastric specimens under harsh denaturing conditions using TRIzol^®^ reagent and dissolved the resulting pellet in 1% SDS.

**Figure 3:**
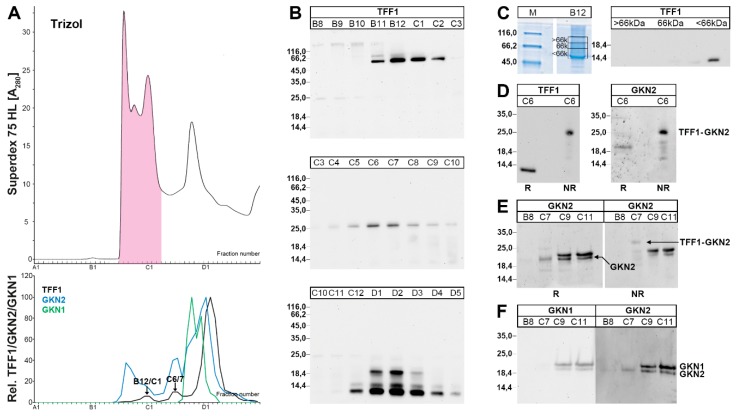
Analysis of a human gastric corpus extract (M_C_-577) after TRIzol^®^ extraction. (**A**) Elution profile after SEC on a Superdex 75 HL column, as determined by absorbance at 280 nm (PAS-positive mucin fractions: pink). Underneath: Distribution of the relative TFF1 (black), GKN2 (blue), and GKN1 (green) immunoreactivities respectively, as determined by Western blot analysis under reducing conditions and semi-quantitative analysis of the typical monomeric band intensities. (**B**) 15% SDS-PAGE (non-reducing conditions) and subsequent Western blot analysis of fractions B8-D5 concerning TFF1 (post-in-gel reduction). The molecular mass standard is indicated on the left. (**C**) 15% SDS-PAGE (non-reducing conditions) and Coomassie staining of fraction B12. Then, the bands >66k, 66k, and <66k were cut out as indicated, proteins were eluted, subject to reducing 15% SDS-PAGE, and the TFF1 content was tested by Western blot analysis. (**D**) 15% SDS-PAGE and subsequent Western blot analysis of fraction C6. Samples were analyzed under reducing (R) or non-reducing (NR) conditions for their TFF1 and GKN2 immunoreactivities, respectively. (**E**) 15% SDS-PAGE and subsequent Western blot analysis of the fractions B8, C7, C9, and C11. Samples were analyzed under reducing (R) or non-reducing (NR) conditions respectively, for their GKN2 immunoreactivity. (**F**) 15% SDS-PAGE (reducing conditions) and subsequent Western blot analysis of the fractions B8-C11 concerning GKN1 and GKN2, respectively.

After SEC of such an extract, TFF1 immunoreactivity was present mainly in three different regions, to different extents (Figure 3): (i) Little immunoreactivity was present in B12/C1 (Figure 3A) with a relative molecular mass of about 60k (Figure 3B). This band contained TFF1, which can be released by reduction (Figure 3C). Thus, this band is expected to represent a yet unknown disulfide-linked TFF1-X heterodimer. (ii) Little TFF1 immunoreactivity was also detectable in the region C6/C7 (Figure 1A), which appeared as a 25k band (Figure 1B). This band contains both TFF1 and GKN2, which can both be released by reduction (Figure 3D). (iii) The major amount of TFF1 peaks in fractions D1-D3 (Figure 3A) and appears in different entities (18k, 14k, <14k).

There was a region from fractions C9-C12, which contained GKN2 as well as GKN1 immunoreactivity, but little TFF1 (Figure 3A, lower panel). Here, a double band appeared in the GKN1/2 range (~19k). In an attempt to clear the nature of this double band, fractions C7, C9, and C11 were analyzed with the anti-GKN2 antiserum (Figure 3E, F) and the anti-GKN1 antiserum (Figure 3F), respectively. Both the anti-GKN2 antiserum and the anti-GKN1 antiserum detected the same double band under reducing conditions in C9 and C11 (Figure 3F). In contrast, in C7, only the lower band was detected by the anti-GKN2 antiserum and this band was missing when using the anti-GKN1 antiserum (Figure 3F). As C7 did not contain GKN1 (Figure 3A, F), the lower band represents GKN2, and this band was shifted under non-reducing conditions to the molecular mass range of the TFF1-GKN2 heterodimer (Figure 3E). The same shift was observed in fraction C6 (Figure 3D).

### 2.4. Characterization of a Human Gastric Extract by Anion-Exchange Chromatography and Protein Identification by Mass Spectrometry

**Figure 4:**
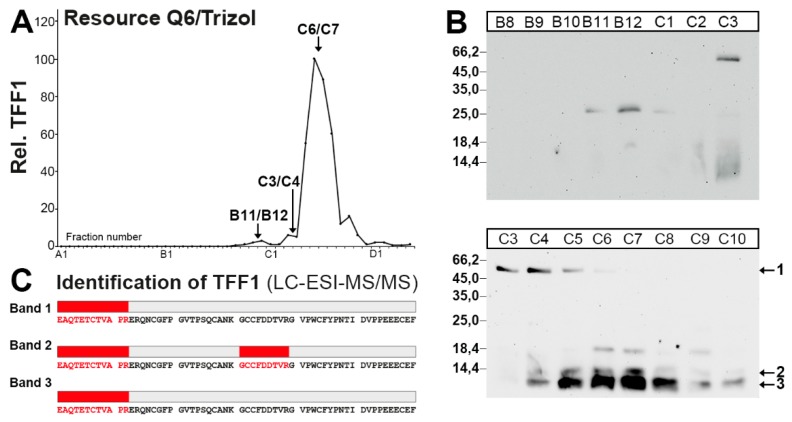
Purification of the 60k-band and monomeric forms of TFF1 from a human gastric corpus extract (M_C_-577) after TRIzol^®^ extraction, and characterization by LC-ESI-MS/MS analysis. (**A**) Anion-exchange chromatography on a Resource Q6 column of a human gastric extract (M_C_-577) after TRIzol^®^ extraction. Shown is the distribution of the relative TFF1 immunoreactivity in the fractions as determined by Western blot analysis under reducing conditions and semi-quantitative analysis of the typical monomeric band intensities. (**B**) 15% SDS-PAGE (non-reducing conditions) and subsequent Western blot analysis of the fractions B8-C10 concerning TFF1 (post-in-gel reduction). Marked are the band at 60k (band 1) and the monomeric bands 2 and 3. The molecular mass standard is indicated on the left. (**C**) Fractions C3/C4 and C6/C7 respectively, were concentrated, desalted, separated by non-reducing 15% SDS-PAGE followed by Coomassie staining, and the regions corresponding to bands 1–3 were excised and subjected to LC-ESI-MS/MS analysis. Shown are the results of the LC-ESI-MS/MS analysis after tryptic in-gel digestion of the bands 1–3. Identified tryptic peptides belonging to TFF1 are shown in red.

In a further attempt to analyze the different TFF1 forms (Figure 1 and Figure 3), a human gastric extract was separated by anion-exchange chromatography (Figure 4A). The 25k TFF1-GKN2 band was clearly detectable in fractions B11/B12 (Figure 4B). Furthermore, the 60k band immunoreactive for TFF1 (designated as band 1 in Figure 4B) was present in fractions C3-C5 and probably monomeric TFF1 forms (double bands < 14k; designated as bands 2 and 3; Figure 4B) were visible in fractions C4-C10.

Bands 1–3 were subjected to tryptic digestion followed by liquid chromatography-electrospray ionization-tandem mass spectrometry (LC-ESI-MS/MS) analysis for protein identification (Figure 4C). Human TFF1 was identified in all three bands.

### 2.5. Binding of ^125^I-TFF1 to Human Gastric Mucin in Vitro (Overlay Assays)

**Figure 5:**
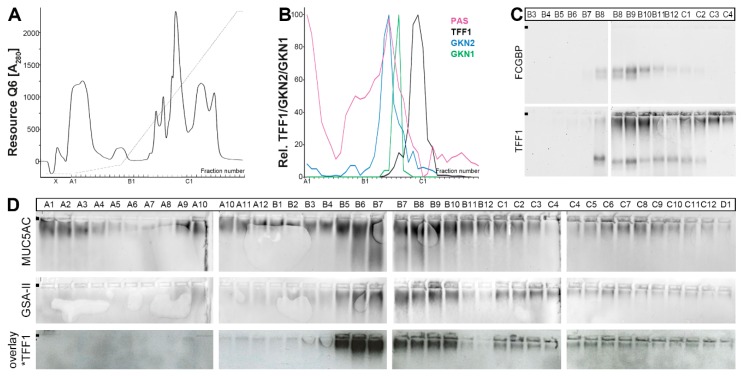
Analysis of human gastric corpus E1-extract (M_C_-577) and binding study with ^125^I-labeled synthetic human TFF1 homodimer. (**A**) Elution profile after anion-exchange chromatography on a Resource Q6 column, as determined by absorbance at 280 nm. Elution was with a salt gradient (dashed line). (**B**) Distribution of the relative TFF1 (black), GKN2 (blue), and GKN1 (green) immunoreactivities respectively, as determined by Western blot analysis under reducing conditions and semi-quantitative analysis of the typical monomeric band intensities. The mucin content (PAS reaction) is shown in pink. (**C**) 1% AgGE and subsequent Western blot analysis of the fractions B3-C4. The start is marked with a dot on the left. Shown are the reactivities for FCGBP and TFF1, respectively. (**D**) 1% AgGE and subsequent Western blot analysis of the fractions A1-D1. Shown are the reactivities for MUC5AC, MUC6 (lectin GSA-II), and the hybridization signals (autoradiography) obtained after incubating the blot with ^125^I-labeled synthetic human TFF1 homodimer (overlay assay), respectively.

TFF1 has been reported to bind as a lectin to a lipopolysaccharide of *H. pylori* [29]. In an attempt to test whether TFF1 might also be able to bind to gastric glycoproteins, e.g., mucins, a gastric extract was separated by anion-exchange chromatography (Figure 5A) and analyzed for the content of the mucins MUC5AC and MUC6 (using the lectin GSA-II; Figure 5D). As a control, the distributions of TFF1, GKN2, GKN1, and FCGBP were also analyzed (Figure 5B,C).

Total TFF1 was hardly associated with GKN2 and mucin (PAS staining; Figure 5B), but it co-eluted with FCGBP in fractions B8-B12 (Figure 5C). Additionally, TFF1 immunoreactivity was detectable in the high-molecular-mass region typical of mucins (Figure 5C).

The in vitro binding study with ^125^I-labeled homodimeric TFF1 revealed a pattern, which strongly resembled in many details the distribution of the mucin MUC6, but not that of the mucin MUC5AC (Figure 5D).

**Figure 6:**
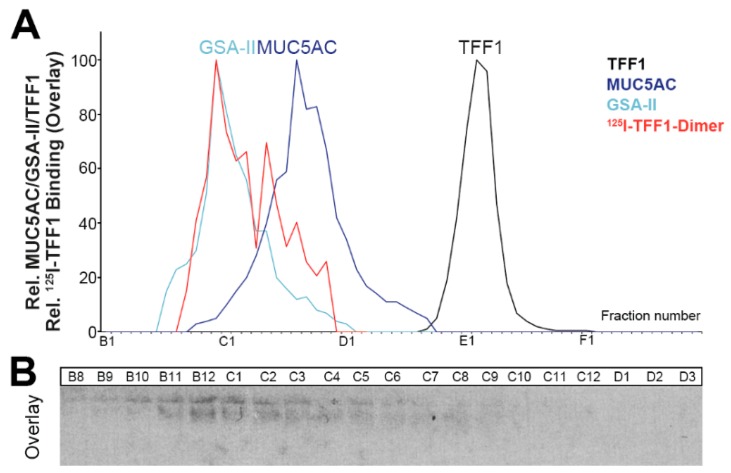
Analysis of a human gastric corpus E1-extract (M_C_-577) after reduction in boiling 1% β-mercaptoethanol and binding study with synthetic ^125^I-labeled synthetic human TFF1 homodimer. (**A**) Distribution of MUC5AC (dark blue), MUC6 (lectin GSA-II; light blue), and TFF1 (black) after SEC on a Sephacryl S-500 HR column. The distribution of MUC5AC and MUC6 on this column has been reported previously [43]. Furthermore, the relative intensity of the hybridization signals (autoradiography and semi-quantitative analysis) obtained after incubating the blot with ^125^I-TFF1 homodimer (overlay assay) is shown in red. (**B**) 1% AgGE and subsequent Western blot analysis of the fractions B8-D3. Shown are the hybridization signals (autoradiography) obtained after incubating the blot with ^125^I-TFF1 homodimer (overlay assay).

To confirm this unexpected result, in vitro binding of ^125^I-labeled dimeric TFF1 was repeated with a human gastric extract separated via SEC on a HiPrep 16/60 Sephacryl S-500 High-Resolution (S-500) column (Figure 6). We have shown previously that this column is able to separate MUC5AC and MUC6 [43]. The distribution of the bound ^125^I-labeled homodimeric TFF1 resembles the distribution of MUC6, but not that of MUC5AC, supporting TFF1 homodimer interaction with MUC6 (Figure 6A).

## 3. Discussion

Generally, multiple forms of TFF1 were recognized in this study, i.e., monomeric and homodimeric forms representing the bulk of TFF1, the TFF1-GKN2 heterodimer (M_r_: 25k), an unknownTFF1 heterodimer with a M_r_ of about 60k (TFF1-X), and a TFF1-FCGBP heteromer. Furthermore, homodimeric TFF1 also binds non-covalently to the mucin MUC6, at least in vitro.

### 3.1. TFF1 Occurs Mainly in Low-Molecular-Mass Forms: Existence of Unusual Monomeric Forms

The bulk of TFF1 appeared in the low-molecular-mass range (Figure 1 and Figure 3), which is in agreement with a previous report [38]. In this range, three entities were detectable, i.e., an 18k band as typical of the TFF1 homodimer and two bands with a M_r_ ≤ 14k, typical of monomeric TFF1. All three entities released monomeric TFF1 after reduction (Figure 1F) and mass spectrometric analysis also confirmed TFF1 in the monomeric bands 2 and 3 (Figure 4C). Furthermore, the presence of a free thiol group was demonstrated by the reaction with PEG-maleimide and the expected shift of the band by a M_r_ of 5k. However, the amount of monomeric TFF1 seems to vary in different individuals (about 50% in Figure 1F and more than 50% in Figure 3B). One possible explanation would be that the disulfide bond of TFF1 homodimers could be cleaved again outside the cell by different mechanisms [44]. In particular, the C*XX*C motif in secretory proteins is known to catalyze disulfide isomerization reactions [44]. Of special note, multiple copies of this motif are present in FCGBP and this protein has already been proposed to play a role in the dimerization of TFF3, which has an analogous structure to TFF1 [45]. This mechanism is of particular interest since FCGBP forms heterodimers with TFF1 and TFF3 [40,45,46].

It is currently not known why monomeric TFF1 appears as a double band (bands 2 and 3) under non-reducing conditions (Figure 1E,F, Figure 3B, and Figure 4B). This could be due to conformational changes of TFF1 [47,48]. As an alternative, a post-translational modification at Cys-58 (e.g., oxidation to a sulfenic acid, etc.) might occur. Of note, in the gastric corpus, band 3 was much more abundant than band 2 (Figure 1F). However, in the gastric antrum, the relative abundance of band 2 was higher than in the corpus (data not shown). Generally, bands 2 and 3 mainly appeared in E0 and E1 (data not shown), indicating that both monomeric TFF1 forms were easily soluble. However, under reducing conditions, a single band was always observed.

The existence of an unpaired cysteine residue is highly unusual for secretory proteins because disulfide formation is enforced in the endoplasmic reticulum (ER) [41]. Thus, TFF1 should be secreted as a disulfide-linked homo- or hetero-dimer, such as TFF1-GKN2, as free thiols act as retention signals for unassembled secretory proteins destined for elimination by the ER-associated degradation (ERAD) system. However, there are examples known, where proteins can escape retention and are secreted in spite of an unpaired thiol group, such as Ig light chains [49]. Here, the retention signal is masked by a flanking acid amino acid residue and transport to the Golgi apparatus can take place [49,50]. A similar situation accommodates to TFF1, where Cys-58 is flanked by even four glutamic acid residues [51]. This sequence is rather unique and found only in two viral proteins [1]. The negative charges might also be a reason why homodimerization of TFF1 is not pronounced. Furthermore, TFF1 produced in *Pichia pastoris* is also mainly secreted in a monomeric form [52] as well as murine Tff1 [40] and the *X. laevis* ortholog xP1 [39]. Thus, the flanking acid amino acid residues around the seventh cysteine residue and monomeric secretion are evolutionarily conserved features of TFF1/Tff1/xP1.

The pKa of cysteine residues can be drastically changed by flanking amino acid residues [53,54]. For example, activated cysteine residues are capable to be preferentially modified, e.g., in response to stress [55]. This could also explain why the intramolecular disulfide bridge in human recombinant TFF1 dimer (via Cys-58) is more prone to reduction than the intramolecular disulfide bridges [35]. Classically, an electropositive environment tends to lower the pKa, stabilizing the thiolate anion and enhancing cysteine reactivity [53,54]. However, this is an oversimplification as the nucleophilicity is not just a simple function of the pKa [50,53,54]. Also, the secondary structure, steric hinderance, and accessibility of the cysteine residue are of great importance [50]. Such a case probably exists at Cys-58 in TFF1, which is of exceptional steric exposure. Cys-58 is situated outside the TFF domain and is even separated by two nearby proline residues (PPEEEC^58^EF). Also, the four negative charges at the flanking glutamic acid residues might increase the nucleophilicity of Cys-58 [54]. This would make Cys-58 an exceptionally reactive cysteine residue, which could have a scavenger function, e.g., for extracellular reactive oxygen/nitrogen species (ROS/RNS).

Such a protection is of particular importance for the stomach, which is a preferred target of ROS and RNS, and might explain why TFF1 is predominantly expressed in the gastric mucosa. This is also in line with the fact that oxidative stress plays a major role for stomach disorders [56]. However, only very little of such a reactive thiolate anion could form in TFF1 in the acid environment (pH 1–2) of the gastric juice [54]. However, TFF1 would be perfectly suited to protect the gastric surface mucous cells and also the adjacent population of highly proliferating precursor cells at the isthmus [6] as these epithelial cells are covered by a mucus-bicarbonate barrier with a pH gradient with near neutral pH at the mucosal surface. The protection of precursor cells is crucial for preventing carcinogenesis and is of particular importance for the gastric antrum because here, the proliferation rate of precursor cells and the turnover rate of surface mucous cells is much higher than in the corpus [5,6].

In the stomach, there is a high level of extracellular ROS, i.e., H_2_O_2_, generated by dual oxidase (DUOX) from the apical surface of gastric epithelial cells, particularly during bacterial infections and chronic inflammatory diseases [57]. Thus, DUOX plays a key role in mucosal immunity preventing, for example, gastric colonization by bacteria [58]. However, this extracellular oxidative stress also damages the extracellular matrix (ECM), which is less well-protected than intracellular sites [59]. For example, the gastric ECM is denuded on a daily basis, e.g., by ingested foods. Such lesions are normally rapidly repaired by migration of surface mucous cells (restitution) [60]. Optimal gastric restitution is heavily dependent on a functional ECM [60]. Of special note, the ECM also forms the niches for stem cells, which need special protection [61]. The free thiol of TFF1 could also well protect extracellular structures from ROS damage.

Furthermore, nitrate (NO_3_^-^) is reduced in the saliva and by microbiota to nitrite (NO_2_^-^) forming instable HO-NO in the gastric juice, which disproportionates into NO, the latter being a highly reactive RNS inducing S-nitrosylation of cysteines [62,63]. This reaction is particularly enhanced by flanking acidic or basic residues [64]. Of special note, S-nitrosylations are catalyzed by copper ions [62], which are known to bind to the glutamic acid residues flanking Cys-58 of TFF1 [65]. Thus, TFF1 is suited to also act as a scavenger for RNS in the acidic environment of the stomach.

Other than being a gastroprotective peptide, TFF1 could also have a protective function during various inflammatory conditions, which are also typical sources for ROS production during the oxidative burst [63]. This might be the reason why TFF1 is ectopically expressed during inflammatory processes in humans as well as in animal models. Typical examples are duodenal ulcers, Crohn’s disease, pancreatitis, asthma, encephalitis, and in the murine spleen after *Toxoplasma gondii* infection [8,9,66,67,68,69].

Finally, the free thiol group of TFF1 could also have a function for the correct assembly of MUC5AC during the secretory process in surface mucous cells. As an indication supporting this hypothesis, the unfolded protein response is activated in *Tff1^KO^* mice [70].

### 3.2. Relatively Little TFF1 Forms a Disulfide-Linked Heterodimer with GKN2 in the Gastric Corpus

When gastric corpus specimens were classically extracted (without SDS) and separated by SEC or anion-exchange chromatography, TFF1-GKN2 was hardly detectable (Figure 1D). Only after extraction with TRIzol^®^ reagent and dissolving the resulting protein pellet in 1% SDS, was TFF1-GKN2 detectable (Figure 3B,D). This has been observed for many humans as well as murine gastric specimens [40]. Furthermore, there are indications that the TFF1-GKN2 content is higher in the antrum when compared with the corpus (data not shown), which would explain why TFF1-GKN2 was straight detected in the antrum [38].

One explanation for the low abundance of TFF1-GKN2 in classical extracts could be that major amounts of TFF1-GKN2 are part of the inner insoluble gastric mucus layer and can hardly be dissolved without SDS. This view would be in line with the observation that total GKN2 accumulated in both the corpus and the antrum in the hardly soluble fraction E2, which was obtained after extraction of the remaining pellet with 1% SDS (Figure 2). First experiments revealed that in E2, GKN2 exists in a monomeric form (mainly in the corpus) and as a TFF1-GKN2 heterodimer (mainly in the antrum). These differences between corpus and antrum could be due to different TFF1/GKN2 expression ratios in the corpus versus the antrum. Furthermore, the expression patterns of TFF1 and GKN2 are not completely congruent, particularly in the corpus [6]. This might prevent the formation of TFF1-GKN2 heterodimers in the corpus and favor the formation of monomeric GKN2.

A role for GKN2 in the firmly attached (inner) layer of the gastric mucus would explain why *Gkn2*-deficient mice have increased susceptibility to *H. pylori*-dependent immunopathology [71]. Thus, GKN2 and TFF1-GKN2 could have a role for stabilizing the inner barrier layer keeping bacteria at a distance. Furthermore, synergistic anti-proliferative and pro-apoptotic effects on gastric cancer cells have also been reported for TFF1-GKN2 heterodimers in the past [72].

### 3.3. TFF1 Forms a Disulfide-Linked Heteromer with an Unknown Partner

When gastric specimens were extracted with TRIzol^®^ and the resulting protein pellet dissolved in 1% SDS, a band with a M_r_ of 60k could be detected after SEC or anion-exchange chromatography (Figure 3B and Figure 4B). Elution of this band and reduction released monomeric TFF1 (Figure 3C), which was confirmed to be TFF1 by mass spectrometric analysis (band 1 in Figure 4C). A similar TFF1 heteromer has already been described in MCF-7 cells before [35]. As a consequence, a partner protein with a M_r_ of about 50k is expected to be disulfide-linked to TFF1. Unfortunately, this protein could not be identified thus far and awaits further characterization.

### 3.4. TFF1 Forms a Complex with FCGBP

In the high-molecular-mass range, a heterodimer with FCGBP was easily detectable (Figure 1B,C and Figure 5C). This is in line with the identification of a TFF1-FCGBP heterodimer also in the murine stomach [40]. Generally, FCGBP was mainly detectable in fractions E0 and E1, indicating that FCGBP is soluble (data not shown). The existence of a TFF1-FCGBP heterodimer in the stomach was not surprising because both TFF1 and FCGBP are secretory products of gastric surface mucous cells and both contain an odd number of cysteine residues [6,51,73]. Furthermore, FCGBP also forms a heterodimer with TFF3, which has an analogous structure like TFF1, including the seventh cysteine residue outside the TFF domain [45,46]. However, the relative amount of TFF3-FCGBP is much higher than that of TFF1-FCGBP. One reason might be the different reactivities of the corresponding cysteine residues in TFF1 and TFF3, respectively (PPEEEC^58^EF versus PLQEAEC^57^TF).

The precise molecular function(s) of FCGBP and the TFF1-FCGBP heterodimer are not known yet. FCGBP is a characteristic protein of various mucin-producing cells and body fluids and is ubiquitous in vertebrates and cephalochordates [73,74]. Its expression is strongly induced by the TH2 cytokine IL-13 [75] and FCGBP is an early response gene after microbial infection [76]. FCGBP is thought to regulate pathogen attachment and disease progression at mucosal surfaces [76] and it is hypothesized to also act as a viral trap for HIV-antibody complexes [77]. Thus, it has to be considered as part of the innate immune defense of mucous epithelia. The multiple modular cysteine-rich domains of FCGBP would be well designed to support a function for the clearing of microorganisms and even a number of bacterial proteins have N-terminal domains homologous to the N-terminal domain of FCGBP [74]. The heterodimerization with TFF1 could synergistically support the binding of microorganisms as TFF1 has a lectin activity, e.g., recognizing a lipopolysaccharide of *H. pylori* [29].

### 3.5. Little TFF1 Binds to Mucin MUC6

Little TFF1 was associated with mucins in vivo (Figure 5C) and the amount bound even had individual variations; for example, in the specimen analyzed in Figure 1B, hardly any mucin-associated TFF1 was detected. This is in line with a previous study [38].

Furthermore, in vitro binding studies (overlay assays) of ^125^I-TFF1 homodimer with a gastric extract fractionated by anion-exchange chromatography also revealed binding to high-molecular-mass mucin fractions (Figure 5D). Of note, hybridization of radioactively labeled monomeric TFF1 (protected at Cys-58 with an acetamidomethyl group) failed to give positive signals (data not shown). The binding pattern with homodimeric TFF1 showed remarkable similarities in some details (particularly in fractions B11 and B12) with the mucin MUC6 (detected by GSA-II binding), but not with the mucin MUC5AC (Figure 5D). However, MUC5AC and MUC6 were not separated from each other on this column.

As we could previously separate MUC5AC and MUC6 by SEC on an S-500 column [43], here, we used just the same fractions for hybridization studies with ^125^I-TFF1 homodimer (Figure 6). The hybridization pattern confirmed that TFF1 bound to the mucin MUC6, but not MUC5AC (Figure 6A). This was surprising because TFF1 is synthesized in surface mucous cells, whereas MUC6 is a secretory product of mucous neck and antral gland cells. Thus, TFF1 seems to interact with the same mucin as TFF2 [43]. These results are in line with a study indicating that binding of TFF1 correlates with binding of *H. pylori* to gastric mucins recognized by the lectin GSA-II [78], i.e., MUC6 [43]. However, binding of TFF1 to MUC6 is in contrast to previous reports describing interactions with MUC5AC [79,80].

Currently, the function of homodimeric TFF1 bound to mucin MUC6 is not known, but it might have a role for stabilizing the two-layered gastric mucus barrier [42]. MUC6 is tightly associated with TFF2 and is less soluble than MUC5AC [43]. Thus, MUC6 seems to fulfill an important role in stabilizing the water-insoluble inner layer [43]. Heterodimeric TFF1 may also be involved here. A future challenge will be to investigate the function of GKN2 for this inner layer because the bulk of GKN2 is concentrated in the hardly soluble fraction E2 (Figure 2). For example, it would be of interest to test whether monomeric GKN2 and/or TFF1-GKN2 can bind to MUC6 as these are the GKN2 forms found in fraction E2.

## 4. Materials and Methods

### 4.1. Human Tissue

All investigations followed the tenets of the declaration of Helsinki and were approved by the Ethics Committee of the Medical Faculty of the Otto-von-Guericke University, Magdeburg (codes: 01/02 January 2002 and July 2007 and 96/06 October 2006). Human specimens from the gastric corpus or antrum were investigated from seven patients (M_A_-250, M_C_-383, M_C_-577, M_A_-679, M_C_-687, M_C_-688, M_A_-690) undergoing gastrectomy because of carcinoma or sleeve resection because of obesity (M_C_-577). Specimens were included in the study only when the formal histopathological review excluded neoplastic changes.

### 4.2. Extraction of Proteins

Extraction of gastric specimens with a 5-fold amount (*w*/*v*) of buffer (30 mM NaCl, 20 mM Tris-HCl pH 7.0 plus protease inhibitors) in a Precellys^®^24 lyser/homogenizer has been described previously in detail [43,81]. Alternatively, the specimens were cut into small pieces, ground with a mortar and pestle under liquid nitrogen, and the fine powder was dissolved in a 5-fold amount (*w*/*v*) of the extraction buffer, similarly as that described previously [45].

Alternatively, 0.8 g tissue was homogenized in a Precellys^®^24 lyser/homogenizer in 8 mL TRIzol^®^ reagent (ambion by Life Technologies, Darmstadt, Germany) and extracted as described previously [43]. The protein pellet was dissolved in 4 mL 1% SDS (2 h at 50 °C).

**Figure 7:**
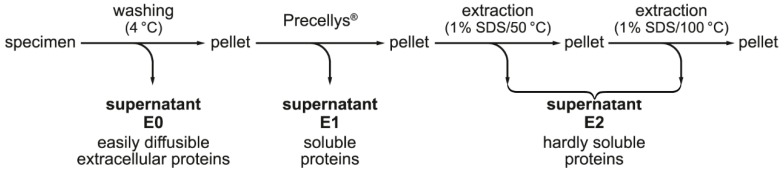
Schematic representation of the stepwise extraction protocol of gastric specimens.

Furthermore, an extended stepwise extraction protocol was applied as described [43]. For details, see Figure 7. In brief, gastric specimens were washed with a 5-fold amount (*w*/*v*) of buffer (30 mM NaCl, 20 mM Tris-HCl pH 7.0 plus protease inhibitors) in a rotating wheel for one hour and after centrifugation, the supernatant E0 was obtained. Then, the remaining cell pellet was extracted in a Precellys^®^24 lyser/homogenizer yielding supernatant E1. Additionally, the remaining cell pellet was dissolved in a 2.5-fold amount of 1% SDS (incubation for 30 min at 50 °C). After centrifugation, the pellet was dissolved again with 2.5-fold amount of 1% SDS (boiling for 5 min). After centrifugation, both clear supernatants after SDS extraction were extracted with CHCl_3_ and combined yielding E2.

### 4.3. Protein Purification by SEC and Anion-Exchange Chromatography

6–8 mL of gastric extracts (or 5 mL after TRIzol^®^ extraction) were fractioned by SEC with the ÄKTA^TM^ FPLC system (Amersham Biosciences, Freiburg, Germany) as described previously (fraction numbering: A1-A12, B1-B12, etc.) [82]. The following columns were used: HiLoad 16/600 Superdex 75 prep grade (S75HL; 20 mM Tris-HCl pH 7.0, 30 mM NaCl plus protease inhibitors; flow rate: 1.0 mL/min; 2.0 mL fractions) or HiPrep 16/60 Sephacryl S-500 High-Resolution (S500; 20 mM Tris-HCl pH 7.0, 30 mM NaCl plus protease inhibitors; flow rate: 0.5 mL/min, 2.0 mL fractions).

Additionally, anion-exchange chromatography was performed as reported previously [45,83] using a Resource Q6 column (Amersham Biosciences; salt gradient from 20 mM Tris-HCl pH 7.0 (buffer A) to 20 mM Tris-HCl pH 7.0 + 1 M NaCl (buffer B); flow rate: 4.0 mL/min, 1.0 mL fractions).

### 4.4. SDS-PAGE, Agarose Gel Electrophoresis, and Western Blot Analysis

Non-denaturing AgGE (containing 0.1% SDS), denaturing SDS-PAGE under reducing or non-reducing conditions, protein staining with Bio-Safe Coomassie Stain G-250 (Bio-Rad Laboratories GmbH, Munich, Germany) without fixation, and periodic acid-Schiff (PAS) staining for mucins (dot blot) were described previously [38,45,82].

Western blot analysis after SDS-PAGE or AgGE was performed as reported in detail [38,43,82,84]. All gels after non-reducing SDS-PAGE were subjected to post-in-gel reduction with 1% mercaptoethanol, as described previously, except for the SDS-PAGE shown in Figure 1D (post-reduction step on the membrane) [82].

The mucins MUC5AC and MUC6 were detected with the polyclonal antiserum anti-MUC5AC-2 (1:200 dilution) [5] and the biotinylated lectin GSA-II from *Griffonia simplicifolia* (2 µg/mL) respectively, as reported [43]. TFF1 was analyzed with the affinity-purified polyclonal antiserum anti-hTFF1-1 (1:1000–2000 dilution) against the C-terminal sequence FYPNTIDVPPEEECEF [85] and GKN2 with the polyclonal antiserum anti-hGKN2-1 (1:3000 dilution) [38]. Affinity purification of polyclonal antisera was previously described in detail [86]. A polyclonal anti-GKN1 antiserum from sheep [87] was kindly provided by Dr. K. Oien (Glasgow). Furthermore, a commercial polyclonal antiserum against amino acids 5176–5344 of the human FCGBP sequence (PAP389Hu01, Cloud-Clone Corp., Katy, TX, USA) was used. Bands were visualized with the ECL detection system and semi-quantitative analysis was performed using the GeneTools software, as described in detail [43].

### 4.5. TFF1 Binding Studies

TFF1 monomer and homodimer were obtained from the Muttenthaler lab and synthesized by solid phase peptide synthesis and native chemical ligation followed by oxidative folding.

Labeling of synthetic TFF1 monomer (protected at Cys-58 with an acetamidomethyl group) and TFF1 homodimer with ^125^I and overlay assays were performed analogous as described previously for TFF2 [83]. In brief, 6 µg TFF1 dimer (1 µg/µL) were labeled with 5 µL Na^125^I (100 mCi/mL, 2000 Ci/mmol; Hartmann Analytic GmbH, Braunschweig, Germany).

### 4.6. Mass Spectrometric Analysis of in-Gel Digested Proteins, Database Searching

Liquid chromatography coupled to electrospray ionization and tandem mass spectrometry (LC-ESI-MS/MS) analysis of in-gel digested proteins was performed as previously described [83].

## 5. Conclusions

TFF1 occurred to a large extent as a monomer with a free thiol, besides forming a homodimer. This is unusual, but in line with similar results for murine Tff1 and the *X. laevis* ortholog xP1. The unpaired thiol group might act as a scavenger for extracellular ROS/RNS, reducing oxidative stress for the gastric mucosa. This could open new clinical perspectives because TFF1 has therapeutic potential, e.g., for reducing mucositis in patients receiving chemotherapy. For example, it would be promising to study whether synthetic peptides mimicking the C-terminal region of TFF1 are able to reduce oral mucositis.

Furthermore, minor amounts of TFF1 form heterodimers with GKN2, FCGBP, and a yet unknown partner with a M_r_ of about 50k. A role for GKN2 and TFF1-GKN2 for stabilizing the inner gastric mucus layer was discussed, whereas FCGBP and TFF1-FCGBP are considered as components of the innate immune defense, probably regulating microbial attachment and clearing of microorganisms. Finally, little TFF1 also bound to the mucin MUC6 and thus might have a role for stabilizing the inner, water-insoluble gastric mucus layer. Taken together, these data are indicative for diverse protective functions of TFF1.

## Abbreviations

AgGE	Agarose gel electrophoresis
FCGBP	IgG Fc binding protein
LC-ESI-MS/MS	Liquid chromatography-electrospray ionization-tandem mass spectrometry
PAS	Periodic-acid-Schiff
SDS-PAGE	Sodium dodecyl sulfate-polyacrylamide gel electrophoresis
SEC	Size-exclusion chromatography
TFF	Trefoil factor family
